# Motivational Factors for Labiaplasty: A Systematic Review of Medical Research

**DOI:** 10.3390/jcm14082686

**Published:** 2025-04-14

**Authors:** Isabel Ortega-Sánchez, María Orosia Lucha-López, Sofía Monti-Ballano

**Affiliations:** 1 Unidad de Investigación en Fisioterapia, Área de Antropología Social, Universidad de Zaragoza, Domingo Miral s/n, 50009 Zaragoza, Spain; iortega@unizar.es; 2 Unidad de Investigación en Fisioterapia, Spin off Centro Clínico OMT-E Fisioterapia SLP, Universidad de Zaragoza, Domingo Miral s/n, 50009 Zaragoza, Spain; smonti@unizar.es

**Keywords:** female genital, cosmetic surgery, motivation

## Abstract

**Background/Objectives**: Labiaplasty is an increasingly popular female genital plastic surgery; however, the motivational factors for women who undergo this procedure are controversial and not fully understood. This review aims to analyze the current medical research addressing the motivational factors behind the increasing prevalence of this procedure. **Methods**: Included studies met the following components: Population (women of any age who have undergone labiaplasty), Exposure of Interest (labiaplasty), and Outcome (motivational factors). The following databases were screened to identify relevant studies: PubMed, Scopus, and Web of Science, using the search strategy (Motivation OR Rationale OR Motive OR Reason OR Incentive) AND Labiaplasty. After analyzing the retrieved studies (*n* = 221), two studies (*n* = 2) strictly met the established eligibility criteria and were included in the results section. One additional study was incorporated after reviewing the reference lists of eligible studies. **Results:** The observed motivations for labiaplasty included purely aesthetic reasons, purely functional impairment, and a combination of both. However, when both factors were considered, functional impairment was typically identified as the primary reason, while concerns about appearance were considered secondary. From a more concrete perspective, it has been noted that negative comments and experiences contribute to the distress surrounding labial appearance. **Conclusions**: The majority of patients undergoing labiaplasty do so for a combination of functional impairment and aesthetic reasons. These motivations may be influenced by external factors. Additionally, there is a scarcity of studies focusing solely on women who have undergone labiaplasty within the broader context of genital cosmetic surgeries.

## 1. Introduction

Female Genital Cosmetic Surgery (FGCS) has experienced significant growth in recent years. FGCS encompasses legally performed procedures aimed at altering the appearance of the genital area without a clear physical or functional medical necessity. These include various types of surgeries, such as labiaplasty, clitoral hood reduction, perineoplasty (tightening of the vaginal opening), vaginoplasty, hymenoplasty, vulvar lipoplasty, and G-spot augmentation [1]. Among these, labiaplasty—a surgical procedure designed to alter the size or shape of the labia minora—is the most commonly requested procedure.

According to the International Society of Aesthetic Plastic Surgeons (ISAPS), the only organization that annually collects such data on a global scale, 189,058 labiaplasty procedures were performed worldwide in 2023, marking a 65.64% increase compared to 2013, the first year this procedure was recorded in the International Survey on Aesthetic/Cosmetic Procedures (ISAPS). These figures may be underestimated as they do not account for procedures performed by gynecologists [2]. Although most labiaplasty requests occur in the private sector, demand in the public sector has also increased, with the number of labiaplasty procedures covered by the United Kingdom’s National Health Service (NHS) quintuplicating between 2001 and 2010 [3]. This rising demand has sparked considerable debate among medical professionals, psychologists, and ethics experts regarding the underlying motivations leading women to seek this intervention.

Labiaplasty is offered as a corrective measure for so-called “labial hypertrophy”. Some medical articles propose specific size thresholds for its diagnosis [4,5,6], even when labia minora measures less than two centimeters [7,8]. Others consider any request acceptable regardless of size, provided it generates concern in the patient, citing physical or psychological discomfort or purely aesthetic motivations [9,10,11]. As a result, this surgery is offered on demand, justified primarily by verbal reports of physical and psychological difficulties, which are not formally assessed [12].

A review of medical literature from the United States and the United Kingdom found that labiaplasty is commonly justified by verbal complaints of vulvar discomfort, including discomfort while sitting or exercising, difficulty wearing underwear or tight pants, chronic infections, hygiene issues during menstruation, psychological distress, sexual difficulties, dissatisfaction with genital appearance, mockery from sisters, or concerns from parents [12].

In general, authors from various studies recognize the coexistence of functional and aesthetic-psychological motivations among women seeking labiaplasty. Some researchers attribute greater significance to functional motivations [13,14,15]. Others, however, note a growing tendency to seek this surgery exclusively for aesthetic reasons [16,17,18,19].

Özer et al. (2018) cautioned that most healthcare professionals perceive the desire for labiaplasty as predominantly arising from dissatisfaction with genital appearance rather than from functional complaints [20]. These authors recommend referring patients for psychological or psychiatric consultation prior to surgery. However, they acknowledge limited evidence regarding the effectiveness of counseling or education in alleviating genital dissatisfaction or enhancing self-esteem [20].

Similarly, Clerico et al. (2017) underscored the relevance of aesthetic motivations, noting that many women requesting labiaplasty may exhibit distorted body image perceptions [21]. These authors question the appropriateness of performing labiaplasty in cases that do not constitute genuine labial hypertrophy with significant functional or psychological implications, further emphasizing that the definition of labial hypertrophy itself remains imprecise [21].

Various studies analyzing information disseminated on popular websites identify three main indications for labiaplasty: functional, aesthetic, and psychological [22].

Functional indications include physical discomfort that interferes with daily life, such as itching, pain during sexual intercourse (dyspareunia), difficulty reaching orgasm due to labia minora inversion, discomfort while exercising (e.g., cycling), irritation or chafing from tight clothing, recurrent urinary tract infections, deviation of the urinary stream, and hygiene difficulties [2,23,24].

Aesthetic indications stem from the desire to enhance genital appearance to conform to a beauty ideal. Surgeons themselves reinforce this aesthetic ideal, promoting the notion that the clitoral hood and labia minora should be small, symmetrical, and concealed within the labia majora [8,25,26]. This preference is colloquially referred to as the “Barbie look” [27,28]. Consequently, many women perceive a “normal” female genital appearance as a completely smooth vulvar surface [29,30,31]. Genital “rejuvenation” is also frequently mentioned, referring to a small, smooth clitoris and prepuce surrounded by small, pink, and concealed labia minora [32].

Psychological indications focus on the emotional impact of genital self-perception, encompassing feelings of insecurity, shame, anxiety, and emotional distress, which become key motivations for surgery. The most frequently cited psychological reasons include a lack of confidence and low self-esteem, social insecurity (e.g., fear of wearing tight clothing or swimwear), shame or insecurity with a sexual partner, affecting sexual life [2,23,24]. Since these concerns are rooted in aesthetic ideals and a perception of genital abnormality, they are often referred to as “psycho-aesthetic” motivations.

Psychological motivations pose ethical challenges, as they may compromise the medical ethical principle of autonomy, which requires that patients make decisions free from psychological coercion. The medical profession has a duty to ensure that women’s consent to medical treatment is valid, meaning that their decision is uninfluenced by external pressures and that no psychological disorders interfere with their choice. If body dysmorphic disorder (BDD) influences the desire for labiaplasty, the validity of informed consent is called into question.

Given the indications cited by medical professionals and their interrelated nature, it is essential to further explore the true underlying motivations leading women to ultimately undergo labiaplasty and how these factors interact.

This review aims to analyze the current medical research addressing the motivational factors behind the growing demand for this procedure. We formulated the following research question: What are the motivational factors in women driving the increasing demand for labiaplasty? Through a systematic evaluation of the literature, this study seeks to provide a comprehensive understanding of the various motivations influencing women’s decision to undergo this procedure, thereby contributing to evidence-based clinical practice and patient-centered care.

## 2. Materials and Methods

The current systematic review was conducted following the guidelines established by the Joanna Briggs Institute, which offers methodologies to provide comprehensive guidance to authors undertaking systematic reviews [33].

The review process adhered to the Preferred Reporting Items for Systematic Reviews and Meta-Analyses (PRISMA) 2020 statement to ensure a clear and systematic report [34].

The systematic review was registered in the International Prospective Register of Systematic Reviews (PROSPERO) (registration date: 24 February 2025). Reference: PROSPERO 2025 CRD420250653374. Available from https://www.crd.york.ac.uk/PROSPERO/view/CRD420250653374 (accessed on 14 March 2025).

### 2.1. Inclusion Criteria

Included studies met the following components: Population (types of participants), Exposure of Interest (independent variable), and Outcome (dependent variable), referred to as PEO. The population refers to women of any age who have undergone labiaplasty. Exposure of interest refers to labiaplasty that involves surgical alteration, usually via reduction of the size of the labia). Outcome refers to the motivational factors. The review included observational studies such as prospective and retrospective cohort studies, case–control studies, analytical cross-sectional studies and qualitative studies.

### 2.2. Exclusion Criteria

Studies that did not meet the PEO components, as well as single case studies, case series studies, reviews, commentaries, consensus, short notes, letters, and articles not available in full text, were excluded.

### 2.3. Information Sources and Search Strategy

The following databases were screened to identify relevant studies: PubMed, Scopus, and Web of Science, using the following search strategy: PubMed and Web of Science: (Motivation OR Rationale OR Motive OR Reason OR Incentive) AND Labiaplasty; SCOPUS: TITLE-ABS-KEY ((Motivation OR Rationale OR Motive OR Reason OR Incentive) AND Labiaplasty), without any filter or limit. Each database was last searched on 13 February 2025. The list of references for all eligible studies was also searched for other relevant studies.

### 2.4. Selection Process

All search results were organized, and duplicate records were removed using Mendeley. The selection process consisted of two phases. Firstly, an analysis of titles and abstracts is needed to identify relevant studies according to eligibility criteria. Secondly, a comprehensive evaluation of the full texts of the selected articles. The selection process was conducted independently by two reviewers (M.O.L.-L. and S.M.-B.), with a third reviewer available to resolve any discrepancies or doubts (I.O.-S.).

### 2.5. Data Collection Process

Two reviewers independently (M.O.L.-L. and S.M.-B.) collected data from each report, with a third reviewer available to resolve any discrepancies or doubts (I.O.-S.).

### 2.6. Data Items

The primary outcome was motivational factors for patients pursuing labiaplasty. A motivational factor was considered to be any reason that patients provided to justify why they had undergone labiaplasty. All results that were compatible with the primary outcome domain in each study were sought for all measures, time points, or types of analyses.

Thus, the motivational factors collected by any method and presented anywhere in the manuscript were considered. The studies also sought participant characteristics.

### 2.7. Study Risk of Bias Assessment

The assessment of the risk of bias was performed using critical appraisal tools from Joanna Briggs Institute (https://jbi.global/critical-appraisal-tools. Last accessed on 6 March 2025). Critical appraisal tools for analytical cross-sectional studies and for qualitative research were selected after analyzing the design of the included studies.

The critical appraisal tool for cross-sectional studies consists of the following eight questions that inquire about aspects that a quality cross-sectional study should include.

1. Were the criteria for inclusion in the sample clearly defined?
2. Were the study subjects and the setting described in detail?
3. Was the exposure measured in a valid and reliable way?
4. Were objective, standard criteria used for measurement of the condition?
5. Were confounding factors identified?
6. Were strategies to deal with confounding factors stated?
7. Were the outcomes measured in a valid and reliable way?
8. Was appropriate statistical analysis used?

The possible responses are Yes, No, Unclear, and Not applicable.

The critical appraisal tool for qualitative research studies [35] consists of the following ten questions that inquire about aspects that a quality qualitative study should include.

1. Is there congruity between the stated philosophical perspective and the research methodology?
2. Is there congruity between the research methodology and the research question or objectives?
3. Is there congruity between the research methodology and the methods used to collect data?
4. Is there congruity between the research methodology and the representation and analysis of data?
5. Is there congruity between the research methodology and the interpretation of results?
6. Is there a statement locating the researcher culturally or theoretically?
7. Is the influence of the researcher on the research, and vice versa, addressed?
8. Are participants and their voices adequately represented?
9. Is the research ethical according to current criteria or, for recent studies, and is there evidence of ethical approval by an appropriate body?
10. Do the conclusions drawn in the research report flow from the analysis or interpretation of the data?

The possible responses are Yes, No, Unclear, and Not applicable.

## 3. Results

### 3.1. Study Selection

**Figure 1:**
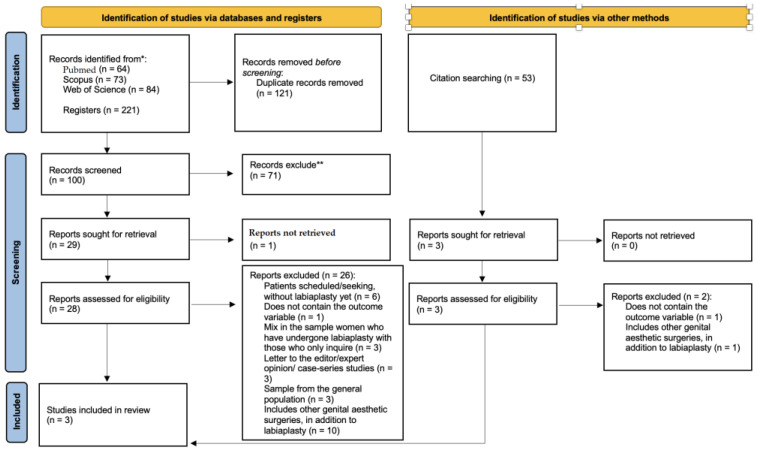
PRISMA 2020 flow diagram. * Consider, if feasible to do so, reporting the number of records identified from each database or register searched (rather than the total number across all databases/registers). ** If automation tools were used, indicate how many records were excluded by a human and how many were excluded by automation tools.

Each database was last searched on 13 February 2025. The number of records identified were PubMed (*n* = 64)/Scopus (*n* = 73)/Web of Science (*n* = 84)/Total (*n* = 221). After searching for duplicates and screening the title and the abstract, *n* = 29 registers were sought for retrieval. One register was not retrieved; thus, *n* = 28 was assessed for eligibility. After analyzing the full text, *n* = 2 studies complied strictly with the established eligibility criteria and were included in the Section 3. One additional study was included after searching the reference lists of eligible studies (Figure 1).

### 3.2. Study Characteristics

Of the three articles finally included in the review, the study by Miklos et al. from 2008 [36] and the study by Rouzier et al. from 2000 [6] were retrospective chart reviews, and the study by Sharp, Mattiske, and Vale from 2016 [37] was a retrospective study based on patient interviews (Table 1).

### 3.3. Risk of Bias in Studies

The study by Miklos et al. from 2008 [36] met five of the eight assumptions required by the critical appraisal tool for analytical cross-sectional studies from Joanna Briggs Institute (https://jbi.global/critical-appraisal-tools. Last accessed on 6 March 2025). It did not meet the two criteria regarding the identification and strategies to deal with confounding factors and the criteria regarding the questionnaire used to measure the motivational factors from the patients. The manuscript did not study confounding factors and did not include data about the validity and reliability of the questionnaire used.

The study by Rouzier et al. from 2000 [6] met the same five of the eight assumptions required by the critical appraisal tool for analytical cross-sectional studies from Joanna Briggs Institute (https://jbi.global/critical-appraisal-tools. Last accessed on 6 March 2025). The manuscript also did not study confounding factors and did not include data about the questionnaire used.

The study by Sharp, Mattiske, and Vale from 2016 [37] met 9 of the 10 assumptions required by the critical appraisal tool for qualitative research studies from Joanna Briggs Institute (https://jbi.global/critical-appraisal-tools. Last accessed on 6 March 2025). It did not meet the criteria regarding the analysis of the influence of the researcher on the research and vice versa. There is no mention or allusion to this issue in the manuscript.

### 3.4. Results of Individual Studies

The characteristics of the participants and the motivational factors addressed in each study are summarized in Table 1.

The study by Miklos et al. from 2008 [36] describes motivational factors related to aesthetic reasons (37%) and functional reasons (32%), with 31% of the sample citing both. Thus, the majority of patients underwent surgery for aesthetic reasons. Although the percentages presented by Rouzier et al. from 2000 [6] are not completely comparable, as in this study, the percentages are not exclusive, and patients could cite multiple reasons, we also observe a higher percentage referring to aesthetic reasons (87%), followed by the percentage related to discomfort when wearing certain garments (64%), discomfort in sports (26%) and invagination of the protuberant tissue (43%), which can be grouped as functional reasons. The study by Sharp, Mattiske, and Vale from 2016 [37], on the other hand, shows the highest percentage in the combination of functional and aesthetic motives (78.6%), followed by functional motives (35.7%) and finally aesthetic ones (21.4%).

The articles by Miklos et al. from 2008 [36] and by Sharp, Mattiske, and Vale from 2016 [37] also investigate potential influences behind the stated reasons. Miklos et al. [36] claim that 93.1% of their sample decided to undergo labiaplasty for purely personal reasons. Although the percentages between both studies cannot be compared due to different study methodologies and data collection modalities, Sharp, Mattiske, and Vale [37], on the other hand, show a high percentage of women (71.4%) who associated with distress related to the appearance of their labia with negative experiences, including negative comments from sexual partners or discussions about the topic in other contexts.

Sharp, Mattiske, and Vale [37] include two relevant percentages in their study that deserve to be mentioned. Fifty percent of their sample reported receiving reassurance or positive comments about their labial appearance; however, this knowledge did not alleviate their concerns about the appearance of their labia. Additionally, 85.7% of the participants were aware that their labia were within the normal size range before surgery, yet they underwent the procedure regardless.

## 4. Discussion

Since the labia minora are two cutaneous folds with a high concentration of sensory nerves, understanding the motivations that lead women to sacrifice healthy and sensitive tissue is essential for healthcare professionals to provide adequate guidance and care to patients considering labiaplasty.

Due to the lack of valid diagnostic criteria for the indication of labiaplasty based on hypertrophy of the labia minora, analyzing the motivations reported by women who have undergone this surgery seems relevant.

The difficulty in establishing a diagnostic criterion derives from the fact that vulvar and vaginal anatomy varies greatly from one woman to another. Previous evidence found [38] that the average width of labia minora in women not seeking labiaplasty was 1.54 cm, compared to 3.52 cm in those who requested it. Previous studies have defined labial hypertrophy as widths greater than 5 cm [4,5]. Consistent with this variability, Motakef et al. [8] found preoperative average labial widths ranging between 2.7 cm and 5 cm, acknowledging that while aesthetic ideals are clearly articulated, there is no consensus across medical specialties—such as plastic surgery, gynecology, and pediatrics—regarding objective clinical criteria for diagnosing labial hypertrophy. In their influential work, Hodgkinson and Hait [9] further highlighted that labia minora protruding beyond the labia majora may be considered aesthetically and functionally unsatisfactory; however, they advised against reducing labial height to less than approximately 1 cm. The study selected in this systematic review from Rouzier et al. [6] established labial hypertrophy as widths greater than 4 cm.

Numerous studies, both retrospective and prospective, have analyzed the reasons why women opt for female genital cosmetic surgery (FGCS) in general [39]. However, few studies differentiate between procedures and specifically examine the motivations of those who have chosen to reduce their labia minora. Additionally, it is common for women requesting labiaplasty of the labia minora to also undergo clitoral hood reduction simultaneously [40,41]. As a result, very few studies have exclusively explored the reasons why women choose to undergo labiaplasty alone. This review aimed to analyze these specific motivations, considering only cases where labiaplasty was performed without additional interventions, such as clitoral hood reduction, which frequently accompanies it.

An analysis of the studies included in this systematic review indicates that aesthetic motivation was the predominant factor for undergoing labiaplasty, although in many cases, it overlapped with functional reasons. In the study by Miklos and Moore [36], 68% of patients cited aesthetic reasons, either exclusively (37%) or in combination with functional reasons (31%), while 32% cited exclusively functional reasons.

Similarly, Rouzier et al. [6] reported that 87% of patients had aesthetic motivations, often accompanied by discomfort associated with wearing tight clothing (64%), engaging in sports activities (26%), or entry dyspareunia due to the inversion of protruding tissue (43%). The study by Sharp et al. [37] found that 100% of women mentioned aesthetic reasons, although a high percentage (78.6%) combined them with functional reasons, generally prioritizing physical concerns as the primary reason for surgery, while aesthetic concerns were secondary. Additionally, 35.7% of participants expressed some skepticism about the necessity of undergoing surgery for purely aesthetic reasons. These findings align with other studies conducted by surgeons on the labiaplasty series, which, while not meeting this review’s inclusion criteria, also reported a predominance of aesthetic motivations, often combined with functional reasons [11,26,42].

None of the studies included in this review explicitly listed psychological motivations, such as insecurity and low self-esteem, in their overall percentages. However, these motivations can be problematic, as the medical ethical principle of autonomy requires that patients make decisions free from psychological coercion, ensuring that no psychological disorders interfere with their choice. If the motivation for labiaplasty is influenced by body dysmorphic disorder (BDD), the validity of informed consent is called into question. The size of the labia minora in women seeking labiaplasty is generally within the published normal limits [3]. Some studies warn that certain women requesting labiaplasty may have BDD. A clinical evaluation is crucial to rule out BDD, as research suggests that cosmetic surgery does not improve—and may even worsen—BDD symptoms, particularly if emotional distress increases significantly due to unsatisfactory aesthetic and functional outcomes [43,44,45]. Although most studies affirm that labiaplasty is a safe procedure with low complication rates and high patient satisfaction [46], a study analyzing online promotion of revision surgery for failed labiaplasties suggests that unsatisfactory results from the consumer’s perspective are fairly common and that the same women whose expectations were not met with the primary surgery are now targeted for a second procedure [47]. Conversely, some studies argue that the harm of denying surgery may be greater than any potential risk from the procedure, particularly in adolescents, where refusal of treatment could be more traumatic and further increase the already high risk of suicide [48].

The interaction between aesthetic, functional, and psychological motivations, as well as their relative importance, remains a subject of debate, as they are often intertwined in each patient’s experience. Although motivations may appear objective, some studies question to what extent functional discomfort is genuinely physical or rather conditioned by psychological factors and aesthetic expectations. Functional discomfort may be influenced by psychological factors derived from the aesthetic ideal. In this sense, a woman’s perception of her genitals, in comparison to the aesthetic ideal, can shape her experience of discomfort. As noted, men rarely complain about physical symptoms associated with genital protrusion, despite having potentially more reasons to do so, nor do they seek to reduce genital mass as a solution [12,22]. This suggests that intolerance to physical sensations in the labia minora is, at least in part, a psychological “discomfort” related to aesthetic dissatisfaction [49].

The idealization of a “normal” genital appearance contrasts with the reality that labia minora size and shape vary widely. One study found that healthy, completely normal labia minora can range from 2 to 10 cm in length, with none of the women in the study expressing any personal or cosmetic difficulty leading them to desire surgical alteration [50]. Another study [3] analyzing the clinical characteristics of women requesting labiaplasty at the gynecology clinic of a London university hospital revealed that all patients presented labial morphology variations that were within normal parameters. This suggests that the sense of disease related to labia minora size is culturally driven and influenced by sociocultural representations of female anatomy.

Additionally, it is likely that many patients emphasize physical difficulties, such as interference with exercise, sexual activity, or wearing tight clothing, as a motivation for surgery since aesthetic dissatisfaction alone might not be considered a sufficient reason for the procedure [51,52]. The study by Sharp et al. [37] found that when aesthetic motivations were combined with functional motivations, the latter were generally presented as the primary reason for surgery, while aesthetic concerns were secondary. Physical or functional difficulties were perceived as more legitimate reasons for undergoing labiaplasty compared to aesthetic concerns.

Many studies argue that concern about aesthetics is not merely a desire for beautification but rather stems from a perception of abnormality based on a specific sociocultural representation of the vulva, in which the labia minora are concealed within the labia majora, despite the actual diversity of anatomical presentations [51,52,53]. The study by Sharp et al. [37] revealed that, despite the vast majority (85.7%) of participants being aware that their labia were within the normal size range and receiving positive feedback, this was not sufficient to change their self-perception or alleviate their aesthetic concerns, leading them to opt for surgery.

Concerns about tight clothing and discomfort during exercise are recurrent themes in the literature on labiaplasty. In the reviewed studies, problems with tight clothing were described both as physical discomfort and psychological distress, the latter linked to shame and anxiety when women perceive their genitals as visible through clothing or swimwear. Documented testimonies illustrate this distress, such as one woman’s embarrassment when a young family member, seeing her naked, commented that she “had a penis” [51]. Another case describes a young woman who rolls up her labia and inserts them into her vagina to reduce the “bulge” [54]. Laufer and Galvin [10] suggest that these situations can negatively impact self-esteem, creating fear that their labia may be perceived as abnormal, leading to questions about their sexual identity and body image. All studies included in this review mention irritation or discomfort caused by clothing as an exclusively physical issue, as well as discomfort during sports activities [6,36,37].

Another recurrent functional concern in the literature on labiaplasty is hygiene. Several studies mention it [7,10,28,32,39,54,55,56]. A study analyzing ten websites from providers of female genital cosmetic surgery in the United States and the United Kingdom found latent associations between labia minora, ugliness, and poor personal hygiene, promoting labiaplasty as a means of improving hygiene [22,52]. Sandra Zwier [52], in her analysis of online forum comments, found that although hygiene was the least frequently cited reason, 7.5% of women mentioned it among their motivations. In this review, hygiene is explicitly mentioned only in the study by Rouzier et al. [6], which indicates that labia minora hypertrophy may be associated with personal hygiene issues, especially during menstruation. In the other two studies [36,37], hygiene was not listed as a primary reason for surgery, although Sharp et al. [37] mention a participant’s testimony stating that her goal with the surgery was to make it “clean and pretty like in pornography”.

The debate on labiaplasty suggests that protruding labia minora may cause sexual dysfunction, as they interfere both functionally and psychologically with women’s sexual activity. Physical or functional barriers include interference during sexual intercourse and dyspareunia, resulting from the involuntary introduction of the labia minora into the vagina during penetration. Psychological barriers include feelings of shame, fear, or insecurity when exposing the genitals. The three studies analyzed in this review agree that labiaplasty is linked to sexuality in two main aspects, although in distinct ways: on one hand, pain or physical discomfort during sexual intercourse [6,36], and on the other, psychological insecurity regarding genital appearance, which affects confidence in intimate situations. Sharp et al. [37] did not document physical problems during sexual activity as motivations among the participating women. However, they did identify high levels of anxiety (85.7%) related to the exposure of their genitalia to their partners. This distress was particularly evident during oral sex and in fear of initiating new sexual relationships.

From a critical perspective, it is suggested that sexual concerns associated with labia minora appearance are linked to the lack of positive cultural symbolization. In contrast, in some African societies, labia minora elongation is a common practice specifically performed to enhance sexual pleasure [57]. This suggests that, due to their rich innervation, labia minora may play a relevant role in arousal and sexual pleasure. However, certain Western discourses assume that a woman with scar tissue surrounding her vaginal opening—and even her clitoris, in cases where labial reduction includes clitoral hood reduction—will experience greater sexual pleasure than a woman with protruding or asymmetrical labia minora.

An essential aspect in the study of labiaplasty is determining whether the motivations that lead women to choose this procedure are strictly personal or influenced by external factors. As observed, concern about aesthetics does not arise spontaneously in an individual vacuum but rather relies on a cultural idealization of the vulva. Functional motivations based on physical discomfort—such as irritation from clothing or discomfort during exercise—can be experienced directly and immediately without requiring a prior cultural model. However, as previously noted, the distinction between functional and aesthetic concerns is not always clear, and some studies suggest that certain functional discomforts may be partially influenced by internalized aesthetic expectations. In contrast, aesthetic—or psycho-aesthetic—motivations involve internalizing an external reference as a point of comparison, which requires some external influence on the perception of genital normality. This distinction is essential for understanding the complex interaction between internal and external factors in the decision-making process regarding labiaplasty, as well as for critically evaluating the reasons behind the increasing popularity of this procedure.

Numerous studies have analyzed the elements or factors that may influence the decision to undergo labiaplasty, highlighting not only negative comments from peers or intimate partners [58,59] but also the influence of social circles, the quality of interpersonal relationships [60], and significantly, the role of some medical sites and mass media. A number of studies question the accuracy of the information and content published on cosmetic genital surgery websites in countries such as the United States and the United Kingdom [22], as well as in Belgium, Canada, Brazil [61], Australia [29,62], and Spain [63]. These studies point out that certain websites pathologize protruding labia minora, labeling normal anatomical variations as abnormalities or defects, and they frequently display “before and after” surgical images that emphasize the “natural” aesthetic outcome of the surgery. Another study assessing the quality of YouTube content on genital surgeries classified it as moderate [64].

Many studies express concerns about the influence of online information, the proliferation of genital imagery on the internet, and easy access to pornography. For many women, their main source of information about genital appearance comes from mass media [65]. The internet is regarded as the most influential factor in shaping and disseminating beauty ideals and in modifying attitudes toward cosmetic surgery in general [66], particularly for adolescents, who use it as a primary source of information regarding sexual and reproductive health [67]. Increased exposure to genital images and explicit films has made women more aware of genital variations [25,26]. This media exposure fosters the internalization of beauty ideals and self-comparison, predisposing women to consider labial reduction [60]. One significant concern is the role of the pornography industry in sexual objectification and the standardization of bodily ideals, which can negatively impact self-esteem and body image, leading to feelings of shame, anxiety, and distress. One study even found a significant correlation between pornography consumption and lower genital self-image and self-esteem [68].

Additionally, a study indicated that “softcore pornography”—where actresses are often selected for having small labia minora, and many have undergone surgical reduction—is the preferred genre among women [69]. This exposure to surgically modified genital images and the self-objectification of women affects perceptions of normality and can lead to genital dissatisfaction. Many women aspire to have the vulva they see in pornography and even bring cut-out images from advertisements or magazines (such as Playboy) to show physicians the vulva they desire [1].

Conversely, some studies conclude that while pornography influences some women’s desire for labiaplasty, its role is relatively minor compared to other motivating factors [59]. Another study found that the most popular pornographic videos featured a variety of genital appearances, though the most viewed or most popular videos may still shape expectations regarding genital aesthetics [70].

Another notable trend linked to labiaplasty is the increasing prevalence of full or partial pubic hair removal. Many medical articles suggest that labiaplasty has emerged as a consequence of the widespread adoption of pubic hair removal. This trend has made previously less visible areas of the vulva more exposed, contributing to a new genital aesthetic ideal where nothing should protrude from the genital area [71]. However, the fact that hair removal increases vulvar visibility is not the cause of the aesthetic ideal. On the contrary, increased visibility should theoretically highlight the diversity of genital morphology. Instead, pubic hair removal increases the pressure on depilated women to reshape their vulva to align with the prevailing aesthetic standard. Therefore, new depilation patterns do not create the ideal but do amplify the pressure to conform, leading some women to choose labiaplasty.

Among the studies analyzed in this review, only two explicitly examined the role of external influences in the decision to undergo labiaplasty. The research by Sharp et al. [37] highlights how the decision to undergo labiaplasty does not occur in an individual vacuum but rather within a network of social expectations, media influences, and interpersonal experiences that shape body perception and normality standards. Their study suggests that the impact of negative comments and peer influence may be much deeper than women openly acknowledge. The study found that 71.4% of participants who underwent labiaplasty had received negative comments about their genital appearance from sexual partners, family members, or even medical professionals. Even though half had also received reassuring or positive messages, and 85.7% knew that their labia minora were within the normal range, these experiences generated distress and reinforced the perception of abnormality, ultimately leading them to choose labiaplasty. Moreover, Sharp et al. [37] emphasized media exposure as a critical factor in shaping these aesthetic concerns, particularly representations of female genitalia in television, aesthetic surgery websites, and pornography.

This review is subject to certain limitations. The first limitation is the small number of studies that exclusively include patients who have already undergone labiaplasty. The literature tends to analyze samples of women seeking information about labiaplasty, either online or at a clinic, without clarifying whether these women ultimately underwent the procedure. Furthermore, although the article highlights motivations for labiaplasty, most of the literature includes women who also received other procedures related to female genital surgery without investigating the justification for those additional procedures. Another limitation of this study relates to the methods used to gather information about the motivations for which the sample underwent labiaplasty in the included studies. In retrospective studies, standardized tools that have been shown to be valid and reliable are not utilized, as they have not been developed. The qualitative research did not analyze the potential influence of the researcher on the research. Despite these limitations, we believe that this study helps clarify the motivations for labiaplasty in women who have undergone the procedure rather than just those who may show interest or curiosity. It restricts the analyzed samples exclusively to this type of surgery, allowing us to draw conclusions specifically for this procedure when it is not performed alongside others that may involve different motivations and functional consequences.

## 5. Conclusions

The majority of patients undergoing labiaplasty do so for a combination of functional impairment and aesthetic reasons. These motivations may be influenced by external factors, such as social expectations, media influences, and interpersonal experiences that shape body perception and normality standards. Notably, there is a scarcity of studies that focus exclusively on women who have undergone only labiaplasty within the broader context of genital cosmetic surgeries. Most retrieved studies involve samples in which, for a number of subjects, other surgical interventions accompany labiaplasty, often including clitoral hood alteration. Although clitoral hood reduction is another elective procedure, it appears that women frequently undergo clitoral hood reduction alongside labiaplasty.

## Figures and Tables

**Table 1:** Summary of individual included studies.

Author, Year	Study Design	Participants Characteristics	Motivational Factors
Miklos et al., 2008 [36]	Retrospective chart review	131 women 35.7 years (mean age, range 14–57) 95% white, 3% African American, and 2% Asian	37%—only aesthetics reasons (8.2%—influenced by their male partners) 32%—only functional ^1^ reasons. 31%—both functional ^1^ and aesthetic reasons (7.5% influenced by male partners and 5.0% by female partners). 93.1%—had purely personal reasons and 6.9% were influenced by a male or female partner or friend.
Rouzier et al., 2000 [6]	Retrospective chart review	163 women 23 years (mean age, range 12–67) Previous size of the labia minora: maximal distance between the base and the edge was greater than 4 cm	87% ^2^—aesthetics reasons. 64% ^2^—discomfort in clothing. 26% ^2^—discomfort in sports. 43% ^2^—invagination of the protuberant tissue.
Sharp, Mattiske, and Vale, 2016 [37]	Retrospective study based on patient interviews	14 adult women 38.4 years (mean age, range 23–59) 100% Caucasian	78.6%—both functional and appearance reasons. 35.7%—to alleviate physical discomfort. 21.4%—only cosmetics reasons. 71.4%—at least one negative experience conditioning their distress surrounding their labial appearance (predominantly negative comments received from former sexual partners, but also discussions with friends, family members, and medical professionals). 50.0%—reassurance or even positive comments about their labial appearance. 85.7%—were aware that their labia were within normal size before surgery.

^1^ 55%—experiencing discomfort while wearing clothing, 46%—discomfort during exercise or activity, and 60%—having painful/uncomfortable intercourse. ^2^ Percentages are non-exclusive; participants could select multiple reasons.

## Data Availability

All data analyzed during this study are included in this published article.

## Abbreviations

The following abbreviations are used in this manuscript:

FGCS	Female Genital Cosmetic Surgery
ISAPS	International Society of Aesthetic Plastic Surgeons
NHS	National Health Service
PEO	Population (types of participants), Exposure of interest (independent variable), Outcome (dependent variable)
BDD	Body Dysmorphic Disorder
